# Effect of screening for Type 2 diabetes on population-level self-rated health outcomes and measures of cardiovascular risk: 13-year follow-up of the Ely cohort

**DOI:** 10.1111/j.1464-5491.2012.03570.x

**Published:** 2012-07

**Authors:** M Rahman, R K Simmons, S H Hennings, N J Wareham, S J Griffin

**Affiliations:** 1 General Practice and Primary Care Research Unit, University of CambridgeCambridge; 2 Northwick Park Hospital, NWLH TrustHarrow; 3 MRC Epidemiology Unit, Institute of Metabolic Science, Addenbrooke’s HospitalCambridge, UK

**Keywords:** diabetes, Ely, mortality, population, screening

## Abstract

**Aims:**

There is continuing uncertainty regarding the overall net benefits of population-based screening for Type 2 diabetes. We compared clinical measures, prescribed medication, cardiovascular morbidity and self-rated health in individuals without diabetes in a screened vs. an unscreened population.

**Methods:**

A parallel-group, cohort study of people aged 40–65 years, free of known diabetes, identified from the population register of a general practice in Ely, Cambridgeshire (*n* = 4936). In 1990–1992, one third (*n* = 1705), selected randomly, received an invitation for screening for diabetes and cardiovascular risk factors at 5-yearly intervals (screened population). From the remainder of the sampling frame, 1705 randomly selected individuals were invited to diabetes screening 10 years later (unscreened population). Patients without known diabetes from both populations were invited for a health assessment.

**Results:**

Of 3390 eligible individuals without diabetes, 1442 (43%) attended for health assessment, with no significant difference in attendance between groups. Thirteen years after the commencement of screening, self-rated functional health status and health utility were identical between the screened and unscreened populations. Clinical measures, self-reported medication and cardiovascular morbidity were similar between the two groups.

**Conclusions:**

Screening for diabetes is not associated with long-term harms at the population level. However, screening has limited long-term impact on those testing negative; benefits may largely be restricted to those whose diabetes is detected early through screening.

## Introduction

Type 2 diabetes meets many of the criteria for suitability for screening [1]. Population screening has been recommended by many national organizations and the National Health Service (NHS) currently includes assessment of risk of diabetes in its Health Checks programme [2]. However, important uncertainties remain about the overall benefits and costs of undertaking population-based screening for this condition, including the impact on the majority with normal screening tests as well as the minority found to have diabetes [3].

Results from ADDITION-Europe, a cluster-randomized trial of intensive, target-driven management of screen-detected patients, suggest that individuals found and treated earlier have a mortality and cardiovascular risk that is lower than clinically diagnosed patients and similar to age- and sex-matched general populations without diabetes [4]. There have also been suggestions of benefit of diabetes screening at the population level. A modelling study from the USA indicated that screening would be cost-effective if started between the ages of 30 and 45 years, with screening repeated every 3–5 years, and would lead to significant reductions in myocardial infarction and diabetes-related microvascular complications in a screened compared with an unscreened population [5]. An examination of the mortality experience of the Ely cohort [6] suggested that individuals who were invited to diabetes screening had a non-significant 21% lower mortality (hazard ratio 0.79; 95% CI 0.63–1.00, *P* = 0.05) than individuals who were not invited to screening between 1990 and 1999.

In addition to impacting on mortality and diabetes-related complications, screening for diabetes may also impact on psychological and behavioural outcomes at the population level. On the one hand, taking part in a screening programme might promote positive behaviour change by sensitizing people about a disease and providing personalized information on individual risk [7,8]. On the other hand, screening may falsely reassure some people, leading to complacency about health and healthy behaviour, particularly among those who screen negative [9,10]. The overall benefits of screening for diabetes should therefore outweigh any associated physical or psychological harm [7]. Recent findings from studies nested in the ADDITION-Cambridge trial [11] suggest that screening does not appear to be associated with psychological harm [12], nor does it falsely reassure individuals with negative results [13].

Effects of screening on self-rated health, a strong predictor of morbidity and mortality, also deserve attention [14]. This measure has often been neglected in screening programmes, in which individual preferences are generally poorly documented and widely variable [15]. Most diabetes screening studies including self-rated health assessment have used small samples and do not always include a comparison with an unscreened (control) group [16–18]. Two diabetes screening studies reported no difference in self-rated health between screening and control groups in a trial at 12–15 months [12] or between those who screened negative and unscreened individuals at 3–6 months or 12–15 months after screening [13]. However, these studies used a single item to measure self-assessed health and focused on the short- or medium-term effects of screening.

In order to examine the effect of diabetes screening on self-rated health and measures of cardiovascular risk at the population level, we compared 13-year outcomes in individuals without diabetes from a screened and unscreened group from the same population.

## Subjects and methods

**FIGURE 1:**
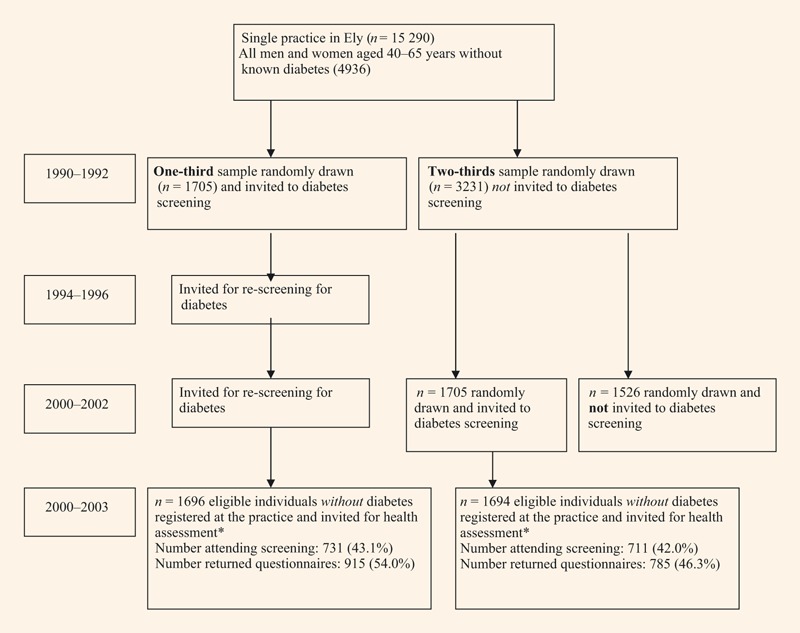
The Ely study population. *Individuals diagnosed with diabetes between 1990 and 1992 and the health assessment in 2000–2003 were excluded from this analysis.

The Ely Study (Cambridgeshire, UK) was established in 1990 as a prospective study of the aetiology of Type 2 diabetes. Full details of the population are reported elsewhere [19]. In brief, a third (*n* = 1705) of all men and women aged 40–65 years were randomly selected from a sampling frame of adults free of known diabetes registered with a single practice serving Ely (*n* = 4936). Housebound individuals were excluded prior to invitation. These individuals were invited between 1990 and 1992 for screening for Type 2 diabetes using a 75-g oral glucose tolerance test and related cardiovascular disease risk factors (the screened population). Further follow-up of this group occurred at a median of 4.5 years (1994–1996) and 10 years (2000–2003), including invitation to non-attenders at baseline (Fig. 1). At each screening round, general practitioners were informed by letter of participants’ fasting plasma cholesterol and triacylglycerol values, blood pressure and the results of the oral glucose tolerance test. Among the remaining two-thirds of the sampling frame who were still alive in 2000–2003, 1577 individuals were randomly selected for invitation to a health assessment, including an oral glucose tolerance test (the unscreened population). No standard intervention package was specified for people found to have Type 2 diabetes or elevated cardiovascular disease risk factors following screening. General practitioners were informed of the results and advised to take whatever action they thought necessary. The Ely study was approved by the Cambridge Local Research Ethics Committee (99/246). All participants gave written informed consent.

Postcodes were available for 90% of participants and were linked to enumeration districts to calculate the Townsend Index—a composite measure of material deprivation based on four factors derived from the 1991 UK census (unemployment, overcrowding, car ownership and home ownership). The index is a standardized *z*-score, which represents local deprivation relative to mean deprivation in England and Wales; a score above 0 implies deprivation is greater than the mean for England and Wales, while a score below 0 indicates less deprivation [20].

### Health assessment visit

In order to assess the potential impact of screening on self-rated health and cardiovascular risk measures, all individuals from both the screened and unscreened populations who were still alive in 2000 received a postal invitation asking them to state whether they had diabetes and if they would agree to attend for a health assessment visit. Invitation was restricted to those still registered at the practice. Those who did not reply were telephoned and invited to attend. Those unable to attend the testing centre were offered a home visit and those unable to visit because of ill health were classified as non-attenders. Those unable or unwilling to attend for testing were asked if they would fill in a postal questionnaire. This analysis excludes individuals diagnosed with diabetes at any testing stage and/or diagnosed clinically.

Participants were asked to attend a local testing centre (Princess of Wales Hospital, Ely) where they underwent an examination that included biochemical, anthropometric and questionnaire measures. Measures were undertaken by centrally trained staff following standard operating procedures. The staff were unaware of study group status.

### Clinical assessments

Blood pressure was calculated as the mean of three measurements taken 1 min apart, while participants were seated with the cuff on the right arm, using an automatic sphygmomanometer (Accutor; Datascope, Huntingdon, UK). Height and weight were measured in light indoor clothing, without shoes, using a fixed rigid stadiometer and a Seca scale, respectively. Participants underwent a 75-g oral glucose tolerance test and a sample of venous blood was taken for assessment of total cholesterol, LDL cholesterol and glycosylated haemoglobin. All biochemical samples were tested at Addenbrooke’s Hospital, Cambridge.

### Assessment of prescription drugs

The self-report questionnaire included items asking about currently prescribed medication.

### Assessment of cardiovascular disease

Cardiovascular disease was assessed using the self-report Rose Angina questionnaire: a score > 3 was defined as the presence of clinical ischaemic heart disease **[**21,22**]**. Participants were also asked if they had experienced a myocardial infarction or stroke, or if they had been diagnosed with angina, hypertension or hyperlipidaemia.

### Assessment of self-rated health

The self-report questionnaire contained the 36-item Short Form **(**SF-36**)** score **[**23**]** and the EuroQol **(**EQ**)**-5D score for assessing functional health status and health utility, respectively **[**24**]**. The SF-36 is a 36-item state health measure with eight domains **(**physical functioning, role—physical, bodily pain, general health, vitality, social functioning, role—emotion, mental health**)**, yielding two summary scores of physical health and mental well-being; higher values indicate higher well-being, 0 the worst and 100 the best. The EQ-5D is a single index utility measure, containing both a 5-level descriptive system, known as the health state, and a visual analogue scale ranging from 0 **(**worst possible health state**)** to 100 **(**best possible health state**)**.

### Statistical analysis

Characteristics of the patients without diabetes were summarized separately according to whether they were in the screened or unscreened populations using means and percentages. Groups were compared using the unpaired *t*-test for normally distributed continuous variables, the Mann–Whitney *U*-test for non-normally distributed continuous data and the χ^2^-squared test for categorical data. All analyses were completed using Stata version 9.0 **(**StataCorp., College Station, TX, USA**)**.

## Results

After excluding those individuals who had been diagnosed with diabetes between 1990 and 1992 and the health assessment in 2000, there were 3390 eligible individuals without diabetes registered at the practice. There were 1696 in the screened population and 1694 in the unscreened population. In total, 1442 (43%) attended for health assessment, with no significant difference attendance between the two groups (43.1% in the screened population vs. 42.0% in the unscreened population; *P* = 0.51). Individuals who attended were younger than those who did not attend (*P* < 0.001), they lived in less-deprived areas (*P* < 0.001) and were more likely to be male (*P* = 0.04) (data not shown). A greater proportion of the screened compared with the unscreened population responded to the questionnaire (54 vs. 46%, respectively, *P* < 0.001). The mean (sd) duration of follow-up from the initial screening test to outcome assessment for the screened population was 12.5 (1.1) years.

The characteristics of individuals without diabetes from the screened and unscreened populations who attended the health assessment visit are shown in the Table 1. The mean age of the participants attending testing was 62.7 (sd 7.4) years and 46% were male. The Townsend score of deprivation for those attending was –1.54 (sd 1.87). Individuals who attended from the screened population were significantly older than those in the unscreened population (63.5 vs. 61.9 years, respectively, *P* < 0.001). The screened group included a larger proportion of women (*P* = 0.002) and who were more deprived than the unscreened group (*P* = 0.03). There was no difference in the proportion of current smokers between populations. Both groups reported consuming similar amounts of alcohol and similar health service usage.

**Table 1:** Characteristics of individuals without diabetes in the screened and unscreened groups of the Ely cohort at the health assessment in 2000–2003

	Screened group (*n* = 731)	Unscreened group (*n* = 711)	*P*-value*
Age (years)	63.5 (7.7)	61.9 (7.0)	< 0.001
Male, *n* (%)	308 (42.1)	358 (50.4)	0.002
Townsend score†	−1.43 (1.98)	−1.65 (1.73)	0.03
Current smoker, *n* (%)	97 (10.6)	92 (11.7)	0.5
Alcohol consumption, units/week‡	3.2 (0.4–10)	3.0 (0.38–10)	0.6
Number visits to general practitioner in previous year‡	2 (1–4)	2 (1–4)	0.1
Number visits to nurse in previous year‡	1.0 (0.5–2)	1.0 (0–2)	0.4
Clinical characteristics			
HbA_1c_, mmol/mol (IFCC)	35 (6)	36 (7)	0.002
HbA_1c_, % (DCCT)	5.4 (0.5)	5.5 (0.7)	0.002
BMI, kg/m^2^	26.9 (4.4)	27.4 (4.8)	0.07
Waist circumference, cm	91.9 (13.1)	94.1 (13.1)	0.002
Systolic blood pressure, mmHg	132 (16)	132 (17)	0.7
Diastolic blood pressure, mmHg	79 (10)	79 (10)	0.99
Total cholesterol, mmol/l	5.6 (1.0)	5.6 (1.1)	0.8
LDL cholesterol, mmol/l	3.5 (0.9)	3.5 (1.0)	0.7
Self-reported medication			
Anti-hypertensive drugs, *n* (%)	230 (25.1)	197 (25.1)	0.98
Lipid-lowering drugs, *n* (%)	69 (7.5)	72 (9.2)	0.2
Anti-platelet drugs, *n* (%)	81 (8.9)	75 (9.6)	0.6
Antidepressant drugs, *n* (%)	52 (5.7)	38 (4.8)	0.4
Anxiolytic drugs, *n* (%)	5 (0.5)	5 (0.6)	0.8
Cardiovascular disease			
Rose questionnaire > 3, *n* (%)	78 (8.5)	53 (6.8)	0.2
Self-reported myocardial infarction, *n* (%)	28 (4.5)	29 (4.3)	0.9
Self-reported stroke, *n* (%)	13 (2.1)	12 (1.8)	0.7
Self-reported hyperlipidaemia, % (*n*)	201 (29.5)	122 (17.7)	< 0.001
Self-reported angina, % (*n*)	51 (8.1)	44 (6.5)	0.3
Self-reported hypertension, % (*n*)	310 (34.8)	251 (32.4)	0.3
Functional status			
Physical health summary score (SF-36)‡	90 (75–95)	90 (75–95)	0.4
Mental health summary score (SF-36)‡	84 (68–92)	84 (68–92)	0.8
EQ-5D score§	0.82 (0.80–0.84)	0.82 (0.81–0.84)	0.8
EQ-visual analogue scale§	78.3 (77.2–79.4)	77.7 (76.5–79.0)	0.9

All values are mean (sd) unless otherwise indicated.

* Groups were compared using the unpaired *t*-test for normally distributed continuous variables, the Mann–Whitney *U*-test for non-normally distributed continuous data and the χ^2^-test for categorical data.

† A composite measure of material deprivation based on four factors derived from the 1991 UK census.

‡ Presented as median (interquartile range).

§ Presented as geometric means (95% CI). Numbers may not add up to total because of (1) missing data for some variables or (2) higher denominator for questionnaire-based measures.

DCCT, Diabetes Control and Complications Trial; EQ, EuroQoL; IFCC, International Federation of Clinical Chemistry; SF-36, 36-item Short Form.

### Clinical characteristics

There were similar values for BMI, blood pressure, total cholesterol and LDL cholesterol between individuals without diabetes from the screened and unscreened populations. HbA_1c_ and waist circumference values were lower in the screened compared with the unscreened group (HbA_1c_ 35 vs. 36 mmol/mol, respectively, *P* = 0.002; waist: 91.9 vs. 94.1 cm, respectively, *P* = 0.002).

### Self-reported medication

The proportion of patients prescribed anti-hypertensive, lipid-lowering and anti-platelet drugs was similar in the two groups. There was also no difference in the proportion of individuals prescribed antidepressant or anxiolytic drugs.

### Cardiovascular disease

There was no significant difference in the proportion of individuals with a score > 3 on the Rose Angina questionnaire between the two populations. Similarly, there were no significant differences in self-reported myocardial infarction, stroke, angina and hypertension. There was a significantly higher proportion of participants with self-reported hyperlipidaemia in the screened compared with the unscreened population (30 vs. 18%, respectively, *P* < 0.001).

#### Self-rated health

Both groups reported identical mental and physical health summary scores on the SF-36 questionnaire. EQ-5D scores were very similar and not significantly different between the two groups.

## Discussion

Thirteen years after the commencement of a population-based screening programme in a general practice, self-rated functional health status and health utility were identical between screened and unscreened groups. Most clinical measures, self-reported medication and cardiovascular morbidity were similar between the two groups. These data and the related data on outcomes among people with diabetes in the Ely study [25] suggest that the benefits of screening may be largely restricted to those whose diabetes is detected and treated early. This is supported by recent results from the ADDITION-Europe trial, where individuals found and treated earlier appeared to have a mortality and cardiovascular risk that was lower than clinically diagnosed patients and similar to age- and sex-matched general populations without diabetes [4].

This is the first long-term follow-up study of self-rated health and measures of cardiovascular risk in a screened vs. an unscreened population. Both screened and unscreened groups reported identical EQ-5D and summary SF-36 scores, which were comparable with a nationally representative sample of healthy individuals [26]. The lack of difference in self-rated health suggests that there is no significant disadvantage to screening over a period of 13 years. Previous research suggests that any negative effects are likely to be short-lived. Marteau *et al*. found no difference in physical well-being in a population-based randomized trial of cardiovascular screening in those offered screening compared with those not offered screening over a 1-year period [27]. More recently, two diabetes screening studies reported no difference in self-rated health between the screening and control groups in a trial at 12–15 months [12] or between those who screened negative and unscreened individuals at 3–6 months or 12–15 months after screening [13].

The Ely screening programme may not have contributed to a reduction in clinical measures and cardiovascular disease morbidity because of ongoing ad hoc opportunistic screening for diabetes and other cardiovascular risk factors at the primary care level in the UK [28,29]. Continuing improvement in the detection and management of cardiovascular disease risk factors in UK primary care [30] may also have diluted any potential effect of this programme. As there was a significant proportion of non-attenders, and those that did attend were younger and less deprived, the ‘healthy volunteer’ effect may also have contributed to the relative lack of difference between the screened and unscreened populations. Indeed, values for BMI, blood pressure and cholesterol were suggestive of a relatively healthy population. The mean values of blood pressure in both groups were similar to the mean values of attenders in OXCHECK, a UK primary-care based study of 35- to 64-year-olds recruited to a randomized trial of health checks in 1989 [31]. HbA_1c_ and waist circumference values were lower in the screened compared with the unscreened group, with both groups demonstrating mean glucose values below the high-risk category for diabetes recently recommended by an expert committee [32]. While the size of effect was small, if such a reduction could be achieved across a whole population, this might have significant cardiovascular benefits [33]. There was a significantly higher proportion of participants with self-reported hyperlipidaemia in the screened compared with the unscreened population. This suggests that the results of cholesterol tests from screening visits may have been fed back to the screened group, and/or that there may have been increased case detection in this group during study follow-up. However, there was no significant difference in the levels of prescribed lipid-lowering medication or cholesterol between the two groups, suggesting that individuals found to have hyperlipidaemia in the screened group were not necessarily offered pharmacological treatment.

No systematic advice was given to individuals who screened negative in the Ely study; education to change behaviour following screening was limited to those diagnosed with diabetes. This may explain the lack of difference in measures of cardiovascular risk between groups, along with the fact that communication and accurate interpretation of diabetes risk status by screened people is challenging. Brief counselling for behavioural change to all invited individuals, irrespective of their screening outcome, may be necessary if screening programmes are to facilitate lifestyle change and therefore influence health outcomes in those screening negative. Our results suggest that screening alone, without provision of other interventions, is unlikely to have a major impact (either beneficial or harmful) on self-rated health or measures of cardiovascular risk among those who screen negative.

### Strengths and limitations

The Ely cohort is a population-based cohort with long-term follow-up. We assessed self-rated health and cardiovascular measures using standardi**z**ed equipment and protocols, and validated questionnaires, with trained staff unaware of study group allocation. Attendance rates for the health assessment were moderate (48%) and similar in the screened and unscreened populations. However, attenders were younger and lived in less**-**deprived areas than those who did not attend. Furthermore, characteristics of attenders from the screened population were different from those of the unscreened population. The screened group were more likely to be female, but they were older and more deprived than the unscreened group, suggesting no clear direction of potential bias. The screened population were more likely than the unscreened population to respond to the questionnaire, which may also have introduced some bias. The study population was almost entirely Caucasian and the practice was less deprived than the average English practice. Our population may therefore have a higher self-rated health than other populations, as well as a different underlying pattern of health**-**related behaviours. Given the likelihood of **‘**healthy volunteer**’** bias in our sample, our findings may not necessarily be generali**z**ed to a population from a less**-**affluent area or with a greater proportion of individuals from ethnic minority groups.

The loss to follow-up because of death or change of practice after the screening programme may have been a source of bias. However, such a possibility is limited as we found no difference between baseline characteristics of patients still registered at their practice and those that had moved away. Further, there were relatively few deaths and between-group differences were non-significant from 2000 onwards [6]. The self-reported outcomes may have been subject to recall bias. However, we used previously validated instruments, which may not be sensitive to small changes because of a low content validity. Generic instruments (SF-36/EQ-5D) may not be sensitive to specific health-related quality-of-life concerns raised by diabetes screening and may have low content validity in this particular context. However, no disease-specific measure could be used as no common disease links our entire population. Furthermore, there is no screening specific health-related quality-of-life instrument independent of the condition of interest. Screening may lead to comparatively small decreases in quality of life, but the decrease may occur across very large numbers of people and so may still be important. The broader spread of items in the generic quality-of-life scales makes them more suitable for detecting an adverse effect of screening rather than the potential benefits [34]. In combining health profiles and utility measures, we attempted to capture as much information as possible concerning possible effects.

## Conclusions

After 13 years of follow-up, measures of self-rated health were identical among individuals without diabetes in a screened compared with an unscreened population. Our results confirm the emerging position that screening for Type 2 diabetes is not associated with long-term harms at the population level. Clinical measures, self-reported medication and cardiovascular morbidity were similar between the two groups, suggesting that, without additional intervention, the benefits may be largely restricted to those whose diabetes is detected early through screening. The benefits of screening might be increased by greater attention to health promotion among those at high risk but with negative tests, and by strategies to improve uptake among older males in deprived areas.

## References

[1] Simmons RK, Echouffo-Tcheugui JB, Griffin SJ (2010). Screening for type 2 diabetes: an update of the evidence. Diabetes Obes Metab.

[2] Department of Health (2008). Putting Prevention First Vascular Checks: Risk Assessment and Management.

[3] Wareham NJ, Griffin SJ (2001). Should we screen for type 2 diabetes? Evaluation against National Screening Committee criteria. Br Med J.

[4] Griffin SJ, Borch-Johnsen K, Davies MJ, Khunti K, Rutten GE, Sandbaek A (2011). Effect of early intensive multifactorial therapy on 5-year cardiovascular outcomes in individuals with type 2 diabetes detected by screening (ADDITION-Europe): a cluster-randomised trial. Lancet.

[5] Kahn R, Alperin P, Eddy D, Borch-Johnsen K, Buse J, Feigelman J (2010). Age at initiation and frequency of screening to detect type 2 diabetes: a cost-effectiveness analysis. Lancet.

[6] Simmons RK, Rahman M, Jakes RW, Yuyun MF, Niggebrugge AR, Hennings SH (2011). Effect of population screening for type 2 diabetes on mortality: long-term follow-up of the Ely cohort. Diabetologia.

[7] Bankhead CR, Brett J, Bukach C, Webster P, Stewart-Brown S, Munafo M (2003). The impact of screening on future health-promoting behaviours and health beliefs: a systematic review. Health Technol Assess.

[8] Strecher VJ, Kreuter MW, Croyle RT (1995). The psychosocial and behavourial effects of health risk appraisals. Psychosocial Effects of Screening for Disease Prevention and Detection.

[9] Stewart-Brown S, Farmer A (1997). Screening could seriously damage your health. Br Med J.

[10] Tymstra T, Bieleman B (1987). The psychosocial impact of mass screening for cardiovascular risk factors. Fam Pract.

[11] Echouffo-Tcheugui JB, Simmons RK, Williams KM, Barling RS, Prevost AT, Kinmonth AL (2009). The ADDITION-Cambridge trial protocol: a cluster–randomised controlled trial of screening for type 2 diabetes and intensive treatment for screen-detected patients. BMC Public Health.

[12] Eborall HC, Griffin SJ, Prevost AT, Kinmonth AL, French DP, Sutton S (2007). Psychological impact of screening for type 2 diabetes: controlled trial and comparative study embedded in the ADDITION (Cambridge) randomised controlled trial. Br Med J.

[13] Paddison CA, Eborall HC, Sutton S, French DP, Vasconcelos J, Prevost AT (2009). Are people with negative diabetes screening tests falsely reassured? Parallel group cohort study embedded in the ADDITION (Cambridge) randomised controlled trial. Br Med J.

[14] DeSalvo KB, Bloser N, Reynolds K, He J, Muntner P (2006). Mortality prediction with a single general self-rated health question. A meta-analysis. J Gen Intern Med.

[15] Black WC, Welch HG (1997). Screening for disease. Am J Roentgenol.

[16] Edelman D, Olsen MK, Dudley TK, Harris AC, Oddone EZ (2002). Impact of diabetes screening on quality of life. Diabetes Care.

[17] Adriaanse MC, Snoek FJ, Dekker JM, Spijkerman AM, Nijpels G, Twisk JW (2004). No substantial psychological impact of the diagnosis of Type 2 diabetes following targeted population screening: The Hoorn Screening Study. Diabet Med.

[18] Adriaanse MC, Dekker JM, Spijkerman AM, Twisk JW, Nijpels G, van der Ploeg HM (2004). Health-related quality of life in the first year following diagnosis of Type 2 diabetes: newly diagnosed patients in general practice compared with screening-detected patients. The Hoorn Screening Study. Diabet Med.

[19] Williams DR, Wareham NJ, Brown DC, Byrne CD, Clark PM, Cox BD (1995). Undiagnosed glucose intolerance in the community: the Isle of Ely Diabetes Project. Diabet Med.

[20] Townsend P, Phillimore P, Beattie A (2004). Health and Deprivation: Inequality and the North.

[21] Rose G, McCartney P, Reid DD (1977). Self-administration of a questionnaire on chest pain and intermittent claudication. Brit J Prev Soc Med.

[22] Rose GA (1965). Ischemic heart disease. Chest pain questionnaire. Milbank Mem Fund Q.

[23] Ware JESK, Kosinski M, Gandek B (1993). SF-36 Health Survey. Manual and Interpretation Guide.

[24] EuroQol (1990). EuroQol—a new facility for the measurement of health-related quality of life. The EuroQol Group. Health Policy.

[25] Rahman M, Simmons RK, Hennings SH, Wareham NJ, Griffin SJ (2012). How much does screening bring forward the diagnosis of diabetes and reduce complications? 12-year follow-up of the Ely cohort. Diabetologia.

[26] Kind P, Dolan P, Gudex C, Williams A (1998). Variations in population health status: results from a UK national questionnaire survey. Br Med J.

[27] Marteau TM, Kinmonth AL, Thompson S, Pyke S (1996). The psychological impact of cardiovascular screening and intervention in primary care: a problem of false reassurance? British Family Heart Study Group. Br J Gen Pract.

[28] Diabetes UK Early Identification of People with Type 2 Diabetes. Position Statement.

[29] Department of Health (2008). Putting Prevention First – Vascular Checks: Risk Assessment and Management.

[30] Doran T, Fullwood C, Gravelle H, Reeves D, Kontopantelis E, Hiroeh U (2006). Pay-for-performance programs in family practices in the UK. N Engl J Med.

[31] OXCHECK (1995). Effectiveness of health checks conducted by nurses in primary care: final results of the OXCHECK study. Imperial Cancer Research Fund OXCHECK Study Group. Br Med J.

[32] International Expert Committee (2009). International Expert Committee report on the role of the A1C assay in the diagnosis of diabetes. Diabetes Care.

[33] Khaw KT, Wareham N, Bingham S, Luben R, Welch A, Day N (2004). Association of hemoglobin A_1c_ with cardiovascular disease and mortality in adults: the European Prospective Investigation into Cancer in Norfolk. Ann Intern Med.

[34] Guyatt GH, Feeny DH, Patrick DL (1993). Measuring health-related quality of life. Ann Intern Med.

